# The exosome journey: from biogenesis to uptake and intracellular signalling

**DOI:** 10.1186/s12964-021-00730-1

**Published:** 2021-04-23

**Authors:** Sonam Gurung, Dany Perocheau, Loukia Touramanidou, Julien Baruteau

**Affiliations:** 1 Genetics and Genomic Medicine, Great Ormond Street Institute of Child Health, University College London, London, UK; 2 Metabolic Medicine Department, Great Ormond Street Hospital for Children NHS Foundation Trust, London, UK

**Keywords:** Exosomes, Extracellular vesicles, Intercellular communication, Targeting, Multivesicular bodies, Tetraspanins, Lipid rafts, Rab GTPases, Endocytosis

## Abstract

The use of exosomes in clinical settings is progressively becoming a reality, as clinical trials testing exosomes for diagnostic and therapeutic applications are generating remarkable interest from the scientific community and investors. Exosomes are small extracellular vesicles secreted by all cell types playing intercellular communication roles in health and disease by transferring cellular cargoes such as functional proteins, metabolites and nucleic acids to recipient cells. An in-depth understanding of exosome biology is therefore essential to ensure clinical development of exosome based investigational therapeutic products. Here we summarise the most up-to-date knowkedge about the complex biological journey of exosomes from biogenesis and secretion, transport and uptake to their intracellular signalling. We delineate the major pathways and molecular players that influence each step of exosome physiology, highlighting the routes of interest, which will be of benefit to exosome manipulation and engineering. We highlight the main controversies in the field of exosome research: their adequate definition, characterisation and biogenesis at plasma membrane. We also delineate the most common identified pitfalls affecting exosome research and development. Unravelling exosome physiology is key to their ultimate progression towards clinical applications.

**Video Abstract**

**Supplementary Information:**

The online version contains supplementary material available at 10.1186/s12964-021-00730-1.

## Background

Extracellular vesicles (EVs) are released by all cells, prokaryotes and eukaryotes, and regulate intercellular communication in health and disease [1, 2]. Exosomes are a subset of EVs that were initially identified as a cellular mechanism to excrete unwanted cellular products [3]. They are now known to play significant roles in cellular communication by transferring functional proteins, metabolites and nucleic acids to recipient cells [4–6]. They influence a broad range of physiological processes such as immune responses [7], tissue repair [8, 9], stem cell maintenance [10], central nervous system (CNS) communication [6] and pathological processes in cardiovascular diseases [11, 12], neurodegeneration [13], cancer [14] and inflammation [15].

Exosomes have generated considerable interest for clinical application as diagnostic biomarkers and therapeutic cargo carriers [16]. Reduced immunogenicity due to their biocompatibility and a bi-layered lipid structure, which protects the genetic cargo from degradation, makes them attractive as therapeutic vectors. Their small size and membrane composition allow them to cross major biological membranes including the blood brain barrier. Production of engineered exosomes is an active research field, which fosters assessment of various therapeutic cargoes, enhancement of target selectivity and optimisation of manufacturing [17, 18]. A major limitation for successful translation remains the difficulty to precisely target the cell type or organ of interest whilst limiting off-target biodistribution. Another concern is the presence of naturally incorporated cellular genetic impurities with potential immunogenicity [18–20]. To circumvent these difficulties, a better understanding of exosome biology in order to improve therapeutic exosome engineering is key.

In this review, we present the most up-to-date knowledge of exosome biology detailing their biogenesis and secretion mechanisms, targeting of recipient cell, uptake and intracellular signalling. Although we acknowledge the complexity of multiple biological mechanisms for secretion and uptake, we highlight the main mechanisms, which could be relevant to exosome engineering for therapeutic applications. We address as well current controversies and common pitfalls impeding exosome research.

## EV classification

**Fig. 1:**
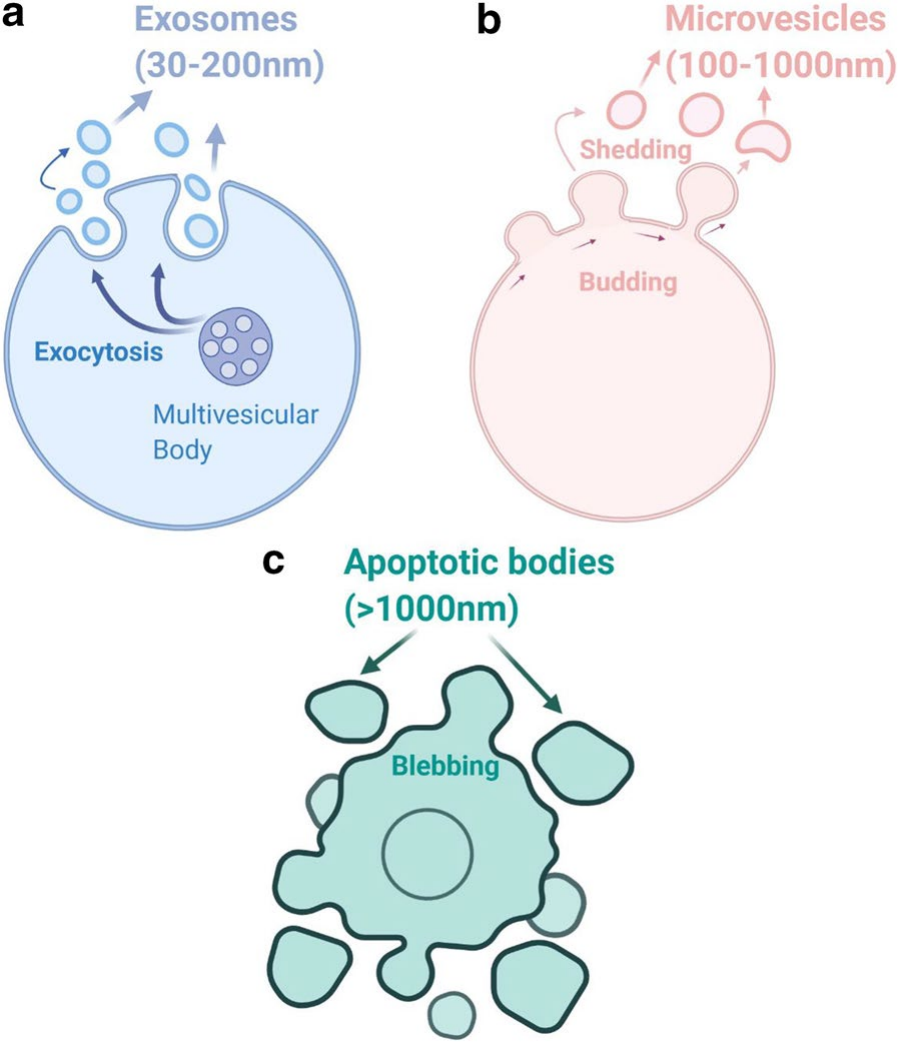
Extracellular vesicles (EVs) classification. The three different classes of EVs are depicted. **a** Exosomes are generated through the endocytic pathway and are released via exocytosis, are spherical in shape and have size range of 30–200 nm of diameter. **b** Microvesicles (MVs) are released through budding from plasma membrane, are irregular in shape and range in size between 100–1000 nm of diameter. **c** Apoptotic bodies are released through blebbing by cells undergoing apoptosis and are > 1000 nm in size

Extracellular vesicles (EVs) are classified into three groups typically based on their size and biogenesis: exosomes (30–200 nm), microvesicles (MVs) (100–1000 nm) and apoptotic bodies (> 1000 nm) [5, 21–24] (Fig. 1). Exosomes are considered to be of endocytic origin, MVs are produced by budding and blebbing from the plasma membrane and apoptotic bodies are released by cells undergoing programmed cell death and signal cell engulfment [25]. EVs are further differentiated based on their density, composition and function [25, 26] (Table 1). While all EVs have complex composition of proteins, nucleic acids, lipids and metabolites (Table 1, Fig. 1), sizes and marker overlaps between heterogeneous EVs can make their differentiation harder [2, 27]. In this review, the exosome terminology is used if clarified in the referenced publication and the term EV is used if the differentiation is unclear.

**Table 1:** Extravesicles subtype characteristics

	Exosomes	Microvesicles	Apoptotic bodies
Origin	Endocytic origin	Plasma membrane budding	Blebbing
Size	30–200 nm	100–1000 nm	> 1000 nm
Density	1.13–1.19 g/ml	1.04–1.07 g/ml	1.16–1.28 g/ml
Shape	Spheroid	Irregular	Variable
Composition	Proteins, nucleic acids, lipids and metabolites	Proteins, nucleic acids, lipids and metabolites	DNA fragments and histone, chromatin remnants, cytosol portions, degraded proteins
Typical constituent proteins	Tetraspanins, ESCRT proteins (Alix, TSG101), integrins, heat shock proteins	Integrins, selectins, CD40 ligand, flotillin-2, adenosine diphosphate ribosylation factor 6, phosphatidylserine	Annexin V, phosphatidylserine
Function	Cell–cell communication	Cell–cell communication	Product of programmed cell death. Facilitate clearance of apoptotic cells
References	[21, 25, 28, 185, 186]	[5, 21, 22, 25, 185, 186]	[21, 23–26]

## Biological composition of exosomes

**Fig. 2:**
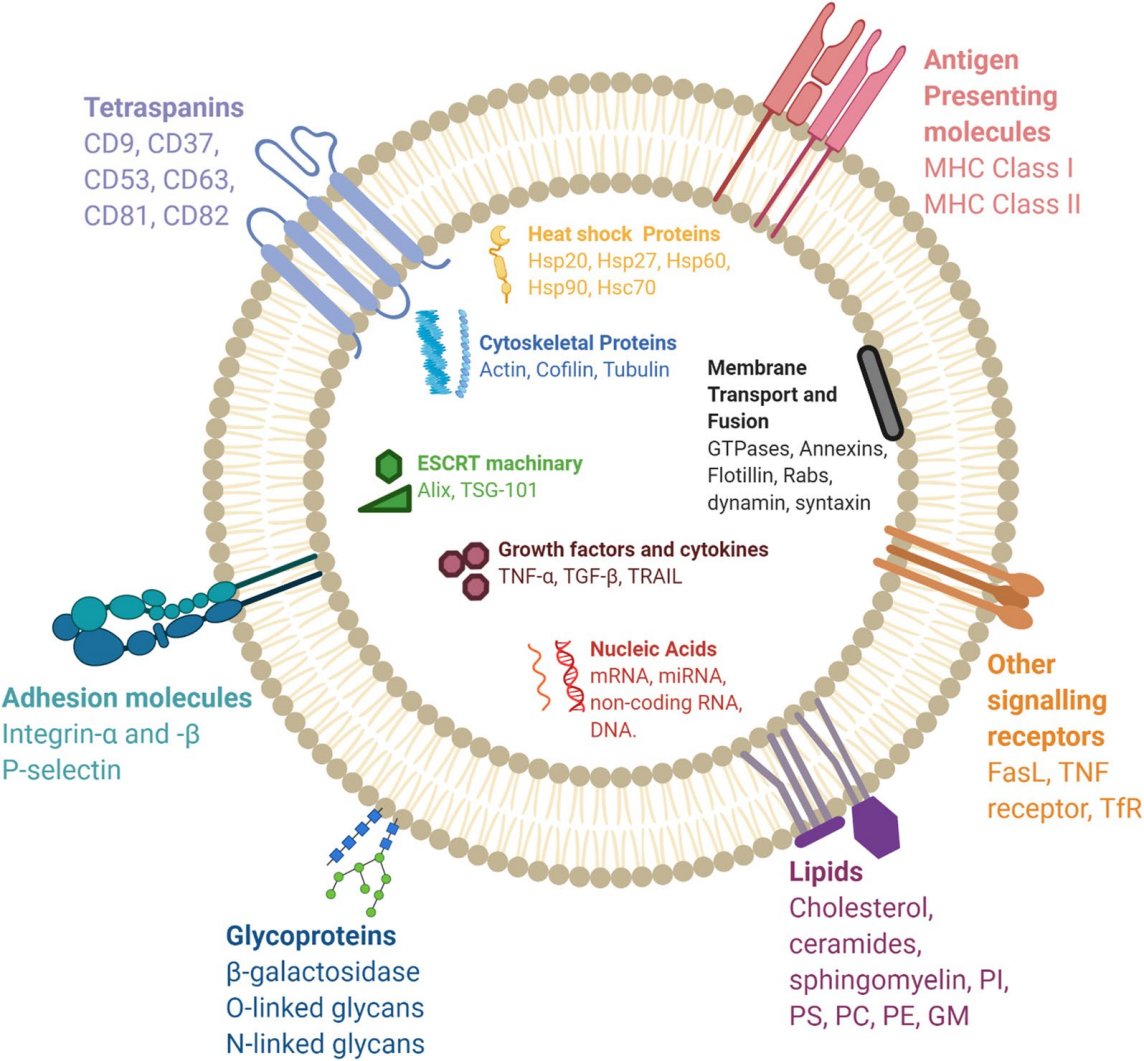
Composition of exosomes. Exosomes are composed of various proteins: transmembrane proteins such as tetraspanins, antigen presenting molecules, glycoproteins and adhesion molecules; proteins in exosome lumen such as heat shock proteins (Hsp), cytoskeletal proteins, ESCRT components, membrane transport, fusion proteins, growth factors and cytokines. Exosomes also comprise of multiple lipids such as cholesterol, ceramides, sphingomyelin, phosphatidylinostol (PI), phosphatidylserine (PS), phosphatidylcholine (PC), phosphatidylethanolamine (PE) and gangliosides (GM) along with nucleic acids such as mRNA, miRNA, non-coding RNA and DNA in their lumen. Hsc = Heat shock cognate; TSG = tumor suspectibility gene; TNF = tumor necrosis factor; TGF = Transforming growth factor; TRAIL = TNF-related apoptosis-inducing ligand; FasL = Fas ligand; TfR = Transferrin receptor

Exosomes are membrane bound carriers. Their cargoes can include proteins, nucleic acids and metabolites (Fig. 2) [28], reflecting the nature of donor cell and its physiological state [12]. Exosomes have a spheroid shape in solution but appear bi-concave or cup-shaped when produced by artificial drying during preparation [29]. The main membrane bound and cytosolic proteins incorporated in exosomes are members of the tetraspanin family (CD9, CD63 and CD81), endosomal sorting complex required for transport (ESCRT) proteins (Alix, TSG101), integrins, heat shock proteins (Hsp), actin and flotillins [16, 30] (Table 2). While proteins such as heat shock proteins, CD63, ESCRT and cytoskeletal components are common among all exosomes, other proteins such as MHC Class I and II are specific to the donor cell type [31]. The rigid bilayer membrane of exosomes also consist of lipid components such as sphingomyelin, cholesterol and ceramides, which influence cargo sorting, exosome secretion, structure and signalling [32, 33] (Table 2). A complex of nucleic acids such as DNA, mRNA and noncoding RNA species as well form part of the exosome composition [2, 31]. MicroRNAs (miRs) are one of the most abundant RNA species in exosomes [16, 34]. MiRs, which play roles in multiple biological processes such as exocytosis, hematopoesis and angiogenesis, participate in exosome mediated cellular communication [16]. Other exosomal RNA species include ribosomal RNA (rRNA), long non-coding RNA (lncRNA), transfer RNA (tRNA), small nuclear RNA (snRNA), small nucleolar RNA and p-element-induced wimpy testis (piwi)-interacting RNA, all of which impact biological processes, particularly influencing tumor development [16, 35]. Studies have thus explored their potential for use as non-invasive disease diagnostic and prognostic tool.

**Table 2:** Exosome composition and roles of main components

Exosome composition			
Category	Examples	Role	References
*Proteins*			
Tetraspanins	CD9, CD63, CD37, CD81, CD82, CD53	Exosome biogenesis, exosome cargo selection, targeting and uptake	[107, 187]
ESCRT machinery/MVB biogenesis	Alix, TSG-101	Exosome biogenesis	[49, 107]
Heat Shock Proteins (Hsp)	Hsp90, Hsc70, Hsp60, Hsp20, Hsp27	Exosomes release, signalling	[188–190]
Membrane transport and fusion	GTPases, Annexins, Flotillin, Rab GTPases, dynamin, syntaxin	Exosome secretion and uptake	[16, 66, 73, 140]
Major Histocompatibility Complex (MHC) molecules	MHC Class I, MHC Class II	Antigen presentation to generate immunological response	[191, 192]
Cytoskeletal proteins	Actin, Cofilin, Tubulin	Exosome biogenesis and secretion	[16, 19, 30]
Adhesion	Integrin-α,-β, P-selectin	Exosome targeting and uptake	[16, 19]
Glycoproteins	β-galactosidase, O-linked glycans, N-linked glycans	Exosomes targeting and uptake	[193, 194]
Growth factors and cytokine	TNF-α, TGF-β, TNF-related apoptosis inducing ligand (TRAIL)	Exosome targeting and uptake, signalling	[116, 162]
Other signalling receptors	Fas ligand (FasL), TNF receptor, Transferrin receptor (TfR)	Exosome targeting and signalling including apoptosis induction and iron transport	[16, 36, 116]

Category	Role	References
*Lipids*		
Cholesterol	Exosome secretion	[195, 196]
Ceramides	Cargo sorting and exosome secretion	[54, 197]
Sphingomyelin	Exosome rigidity and signalling	[111, 197]
Phosphatidylserine	Exosome formation, signalling and uptake	[32, 196, 198]
Phosphatidylcholine	Exosome formation and structure	
Phosphatidylethanolamine	Exosome formation and structure	
Phosphatidylinositol	Exosome formation and structure	
Gangliosides	Exosome rigidity	

## Exosome biogenesis in multivesicular bodies

**Fig. 3:**
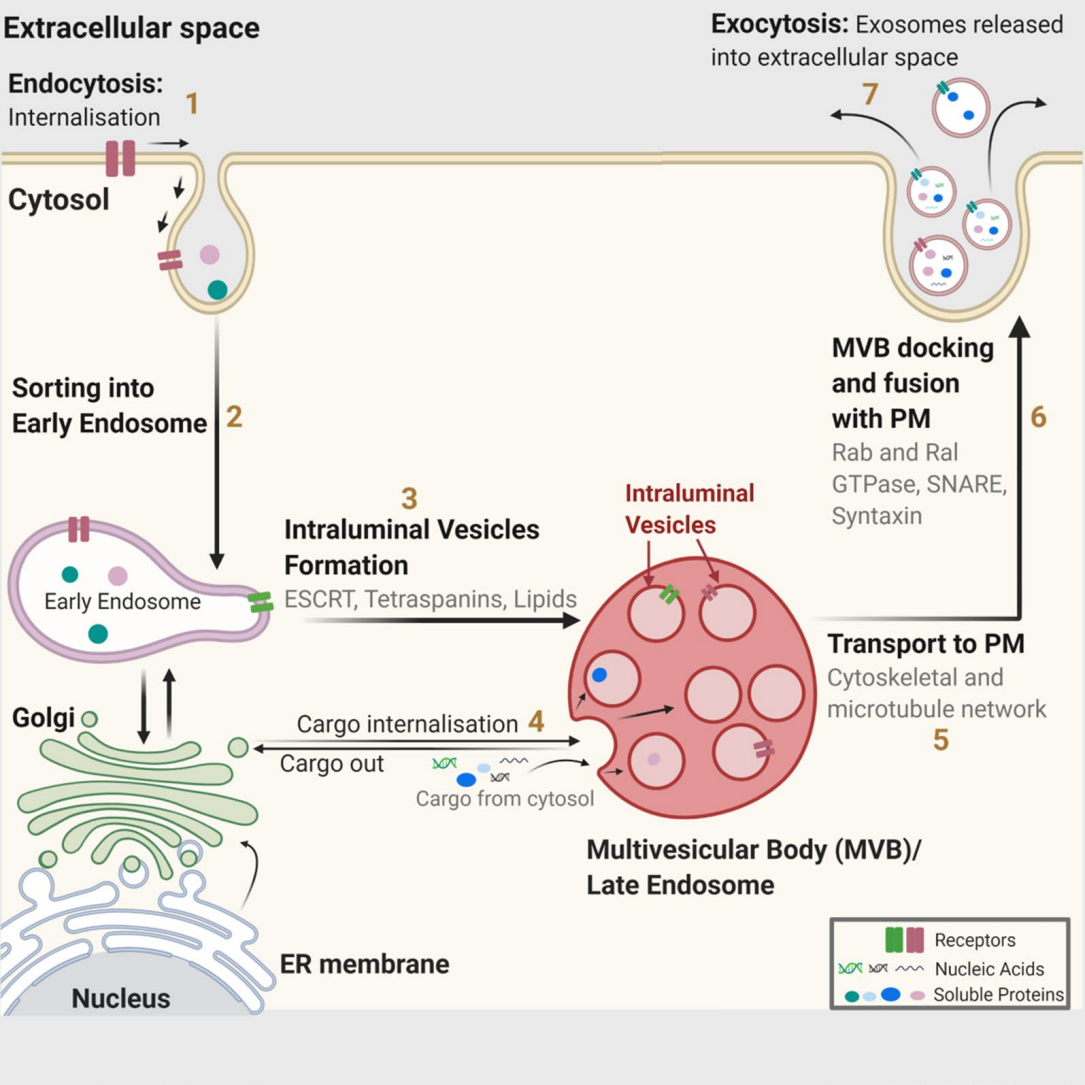
Exosome biogenesis. Within the endosomal system, [1] internalised cargoes are [2] sorted into early endosomes, [3] which then mature into late endosomes or multivesicular bodies. Late endosomes/multivesicular bodies are specialised endosomal compartments rich in intraluminal vesicles (ILVs), which sequester proteins, lipids, and cytosolic compartments and potential exosome cargoes. [4] Cargoes are also delivered from trans-Golgi network and possibly from cytosol. [5] Multivesicular bodies containing exosome cargoes get [5] transported to the plasma membrane, [6] fuse with the cell surface and [7] the ILVs then get secreted as exosomes. ER: Endoplasmic Reticulum; MVB: Multivesicular Bodies; PM: Plasma membrane

Multivesicular bodies (MVBs) and late endosomes are a subset of specialised endosomal compartments rich in intraluminal vesicles (ILVs), which sequester specific proteins, lipids and cytosolic components. Secreted ILVs become exosomes. ILVs are generated by the inward budding of endosomal membranes, first discovered through the study of vesicular secretion of transferrin receptor (TfR) by mature reticulocytes [36]. MVBs get transported to plasma membrane via cytoskeletal and microtubule network and undergo exocytosis post fusion with the cell surface whereby the ILVs get secreted as exosomes [25]. Other MVBs follow a degradation pathway either by direct fusion with lysosomes or by fusion with autophagosomes followed by lysosomes [37]. MVBs are a heterogeneous population [38] and speculation remains whether the secretory and degradatory MVB pathways are distinct. It is also unknown if some specific markers or cargoes influence these pathways. To date, multiple mechanisms involved in exosome biogenesis have been identified. ESCRT machinery plays a prominent role in this process, with SNARE proteins and their effectors such as RAB GTPases playing important role in their secretion alongside [5, 31]. Furthermore, the importance of mechanisms relying on tetraspanins and lipids cannot be underestimated and have helped improve our understanding of dynamics of exosome generation and release (Fig. 3).

ILV biogenesis and secretion are mainly driven by the ESCRT machinery, a cytoplasmic multi-subunit system essential for membrane remodelling, which enables vesicle budding and cargo sorting in MVBs and relies on five core ESCRT complexes: ESCRT-0, -I, -II, -III and Vps4 [39]. Ubiquitinated cargoes are recognised and sorted by the key subunit hepatocyte growth factor-regulated tyrosine kinase substrate (Hrs) of ESCRT-0 to phosphatidylinositol-3-phosphate (PI3P) enriched endosomal compartments [39]. PI3P is a phospholipid found predominantly in early and late endosomes regulating cell signalling and membrane trafficking [40]. In the ESCRT pathway, PI3P promotes cargo organisation through Hrs interaction. Subsequently ESCRT-0 recruits ESCRT-I by interacting with the ESCRT-subunit tumor susceptibility gene 101 (Tsg101). ESCRT-I along with ESCRT-II promotes endosomal inward budding around clusters of ubiquitinated proteins. The charged multivesicular body protein-6 (CHMP6) subunit from ESCRT-III then binds to ESCRT-II and recruits CHMP4 which polymerises as a coil around the neck of the budding ILV pouch. Upon addition of CHMP3, the bud is cleaved forming ILVs, followed by ESCRT-III disassembly using ATP catalysed by Vps4 [39].

Multiple evidences support the critical role of ESCRT in exosome biogenesis through ILV formation. Loss of Hrs, ESCRT-0 subunit STAM1 (Signal Transducing Adaptor Molecule) and Tsg-101 all reduce exosome secretion in multiple cell types such as tumor and dendritic cells [41, 42]. Leptin, a hormone which regulates energy balance and hunger, enhances exosome release by increasing TSG-101 expression [43]. Hepatitis C virus (HCV) infected cells are dependent on CHMP4B (ESCRT-III component) for exosome-mediated transfer of viral RNA [44]. ESCRT components, TSG101 and ALIX, are commonly occuring exosome constituent proteins [16]. ALIX is an accessory ESCRT protein which binds ESCRT-III subunits and aid the budding and abscission process for ILV formation, and is shown to have prominent role in exosome formation particularly in tumor cells. ALIX interacts with syndecan heparan sulfate proteoglycan through its cytoplasmic adaptor, syntenin to drive ILV formation and hence exosome production [45]. The ALIX-syndecan interaction also influences the sorting of syndecan interactor cargoes into ILVs [45–48]. This syndecan-syntenin interaction is also exploited by oncogenic Src kinase in tumor environment to induce exosomal promigratory activity [48]. ALIX also facilitates incorporation and secretion of tetraspanins into exosomal membrane by directly recruiting ESCRT-III to late endosomes [49]. ESCRT-III is recruited through direct interaction with lysobiphosphatidic acid (LBPA), independent of the classic ESCRT pathway [49]. However, in non-tumor cells such as dendritic cells, ALIX silencing increased MHC-II exosomal secretion but reduced CD63 presence in exosomes [41].

ESCRT is a ubiquitination dependent process and ubiquitin binding ESCRT proteins like Hrs, STAM1 and TSG101 all play important roles in exosome biogenesis. However the role of ubiquitination in exosome cargo sorting is unclear. Although ubiquitinated soluble cargoes are enriched in exosomes [50, 51] and presence of ubiquitination sequence in cargoes such as Major Histocompatibility Complex (MHC)-II enhances their ILV incorporation [52], non-ubiquitinated MHC-II are still recovered in exosomes [53] suggesting ubiquitination independent exosome incorporation to also occur.

Alongside ESCRT dependent processes, the roles of complex lipids and other protein related pathways in exosome generation have also been highlighted [33]. Complex lipids such as ceramide can self-associate to form raft-like structures and contribute to the initial membrane curvature for inward budding to form intraluminal vesicles [54]. Loss of sphingomyelinase, an enzyme which breaks down sphingolipid to ceramide, impairs exosome secretion of Aβ- peptides in neurons [55] and exosomes containing CD63, CD81, Tsg101 and miRNAs in multiple tumor models [42, 56, 57]. Sphingomyelinase inhibition also reduces exosomal viral RNA transfer from hepatitis C infected cells [44]. Similarly, Zika virus relies on sphingomyelinase activity in cortical neurons to mediate infection and viral transmission through exosomes [58]. Curcumin, a hydrophobic polyphenol found in the plant *Curcuma longa* and the main compound of turmeric, also drives exosome secretion by increasing intracellular concentration of ceramide and reducing lipid concentration within endolysosomal compartments [59]. ESCRT-dependent and ESCRT-independent lipid-mediated pathways co-exist in numerous biological processes like in the viral RNA transfer and invasive process of carcinoma cells [42, 44]. However, lipid dependent regulation of exosome biogenesis is cell type dependent, for instance in melanoma cells where exosome production is unaffected by the loss of ceramide production [60].

Tetraspanins are highly conserved membrane integral proteins, which play important roles in protein scaffolding and anchoring in cellular membranes [61]. Tetraspanins CD9, CD63 and CD81 are highly present in exosomes, are often used as exosome biomarkers and can influence exosome biogenesis and composition [61–63]. CD63 (LAMP-3) regulates exosome loading of the latent membrane protein 1 (LMP1), the main Epstein Barr Virus (EBV) related oncoprotein, which enables escape from lysosomal degradation [64]. CD63 interacts with apolipoprotein E to regulate loading of premelanosomes and ILV sorting during melanogenesis independently of ESCRT [60, 65]. Interestingly, tetraspanins influences cargo sorting for release or degradation as CD63 loss results in ESCRT-dependent lysosomal degradation of premelanosomes [60]. CD63 is one of the main proteins used in engineered exosomes to faciliate increased loading of cargoes and reporters [6, 66–69]. Other tetraspanins that play roles in exosome biogenesis include CD9 which interacts with metalloproteinase CD10, a common leukemia antigen, to enhance exosomal loading of CD10 [70] and CD81-enriched microdomains provide platform for cargo sorting [63]. Tetraspanin-mediated exosome biogenesis closely interact with complex lipids like the interplay of CD9 and CD82 with ceramide to secrete in exosomes β-catenin, a key protein in cell–cell adhesion and gene expression [56]. Contrastingly, exosome production is negatively regulated by tetraspanin-6, which through its interacting partner syntenin influences ALIX-syndecan-syntenin function and directs MVB cargoes for lysosomal degradation [71].

In terms of exosome secretion, Rab GTPases, the most abundant family of proteins in Ras superfamily of GTPases, play a crucial role in intracellular vesicle transport including endosome recycling and MVBs trafficking to lysosomes [72]. Rab GTPase modulation of exosome secretion is heteregenous, depending on cell-type and cargoes. Rab GTPases and SNARE proteins interact to induce exosome secretion [5]. Rab27a is involved in the MVB docking at plasma membrane in Hela cells [73], neurons and podocytes [74] and in exosome-mediated invasiveness of carcinoma cells [42]. Rab27a also determines exosome size while Rab27b, which shares common function with Rab27a in endosomal trafficking, instead influences the intracellular distribution of MVBs in exosomal trafficking [73]. Rab11 and Rab35, which typically acts in the endosome recycling pathway [75, 76], also influence exosome cargo secretion [76]. Rab11 is required for the exosomal secretion of evenness interrupted (Evi) within the neuromuscular junction of drosophila, which facilitates synaptic development and plasticity [77]. Rab35 is required for the exosomal secretion of myelin protein proteolipid (PLP) in oligodendrocytes [78, 79] by docking MVBs to plasma membrane. Loss of both Rab11 and Rab35 results in enhancement of intracellular accumulation of endosomal cargoes, highlighting their important roles in the cargo secretion and also in recycling of late endosomal compartments [76]. Rab7, which regulates endosomal trafficking of MVBs to lysosomes [78], display contrasting role in exosome release dependent on the cell-type [45, 73, 80]. Additionally Rab2b, Rab5a and Rab9a enhance exosome secretion [73]. Hence, modulation of exosome secretion by Rab GTPases depends on both their distinctive trafficking functions and the cell-type. RAL1 (Ras related GTPases homolog) also mediates ILV budding and tethering of MVB to plasma membrane in breast cancer cells and Candida elegans [81]. RAL1 regulates exosome secretion in co-ordination with T-SNARE syntaxin 5 and an unidentified V-SNARE [81]. R-SNARE Ytk6 interacts with ESCRT-dependent exosome secretion of active Wnt and hedgehog signalling proteins in drosophila and vertebrate cells [82, 83]. Study in HeLa cells also show MVB fusion to plasma membrane and subsequent secretion of exosomes regulated by T-SNARE SNAP23 and syntaxin-4 [66]. As evident, the mechanisms of exosome biogenesis and secretion are heterogeneous with the main ESCRT pathway being seconded by other mechanisms involving lipid rafts and tetraspanins. Rab proteins further aid the cargo sorting and exosome secretion.

Finally, autophagy related protein-5 (Atg5) and autophagy-related-16 like-1 (Atg16L1) also regulate exosome biogenesis in breast cancer cells [84] and mediate exosome secretion of prion proteins in central and peripheral neuronal cells [85]. Autophagy is a regulated self-degradative process that removes unnecessary and dysfunctional cellular components for recycling [86]. Interestingly mechanistic target of rapamycin complex 1 (mTORC1), a highly conserved Ser/Thr kinase and master regulator of autophagy, also negatively regulates exosome release in response to changes in nutrient and growth factors, in a manner similar to autophagy [67]. The concurrent regulation of these two processes likely allows cellular waste management and recycling, particularly under stress conditions. Such regulation of exosome release possibly occurs in stressful environment of glucose starvation and hypoxia [30].

Hence exosome biogenesis is a finely tuned and reactive pathway with multiple molecular players which are involved in other key cellular functions or vesicle related physiology.

## Transport and biodistribution

Exosomes mediate cell–cell communication locally and systemically and are secreted by most cell types including dendritic cells, macrophages, cancer cells and mesenchymal stem cells [87]. Exosomes are present in various biological fluids such as breast milk, blood, serum, urine, saliva, amniotic and synovial fluids [88]. Moreover exosomes might undergo multiple cell uptake and release cycles to allow access to several layers of tissues [89]. Biodistribution studies are commonly performed using heterologous exosomes delivered through various routes of administration, which are likely to behave differently than autologous exosomes, thereby influencing their kinetics and biodistribution [87]. Study using both cell-derived and body fluid-derived exosomes (like bovine milk-derived exosomes) show biodistribution to most organs including liver, lung, kidney, pancreas, spleen, ovaries, colon, and brain after oral administration but an intravenous administration causes a predominant sequestration in the liver followed by spleen, lungs and the gastrointestinal tract [87, 90, 91]. Intravenous injection results in rapid clearance of exosomes in the bloodstream while intratumoral injection allows longer exosome detection in tumors [92] and intranasal administration favors delivery to the brain [17, 93]. Macrophages commonly mediate the uptake in most tissues, while endothelial cells preferentially mediate the uptake in lungs [94–96]. Exosome size also influences transport and biodistribution as larger EVs preferentially accumulate in bones, lymph nodes and liver [97].

Although a non-specific uptake is shared by all cell types [98], specific targeting to recipient cells is paramount to deliver exosome content and exert its function [99]. This is mediated by the surface composition of the exosome (Table 2). For instance, integrating central nervous system-specific rabies viral glycoprotein (RVG) [100], which specifically interacts with acetylcholine receptor enables exosome delivery to the brain [101, 102]. Another exosome targeting specificity is the conservation of tropism between donor and recipient cells. This cellular signature conserved in the secreted exosomes acts as recognition motifs for uptake by the same recipient cell types in vitro and in vivo [103, 104]. For instance cancer cells target cell types by harbouring mannose- and sialic acid- enriched glycoproteins on exosome surface like ovarian cancer cells [105]. Integrins α_6_β_4_ and α_6_β_1_ target lung metastasis and integrins α_v_β_5_ target liver metastasis [106, 107]. Neuroblastoma cells release CD63 positive exosomes targeting neuronal dendrites and CD63 negative exosomes targeting whole neurons and glial cells simultaneously [108]. Equally, the presence of certain receptors facilitates evasion from the host immune system. For instance, CD47 at the surface of engineered exosomes contributes to evasion from host immune cells during circulation in vivo [109]. Complex lipids also influence exosome targeting as observed in cancer cells. Glioblastoma-derived exosomes enriched with phosphatidylethanolamine preferentially target glioblastoma cells along with fibrosarcoma and breast cancer cells [110]. Sphingomyelin enriched melanoma derived exosomes show enhanced targeting in the tumor microenvironment [111]. Lipid targeting is also used by other cell types such as dendritic cells where reduced sphingolipid composition negatively regulates their exosomes uptake ability [96]. Phosphatidylinositol-enriched exosomes decrease macrophage targeting [112]. Therefore cell origin, route of administration and exosome composition are all important factors influencing exosome biodistribution.

## Reaching the recipient cell

When reaching the target cell, exosomes can either trigger signalling by directly interacting with extracellular receptors or be uptaken by direct fusion with the plasma membrane or get internalised.

### Direct interaction

**Fig. 4:**
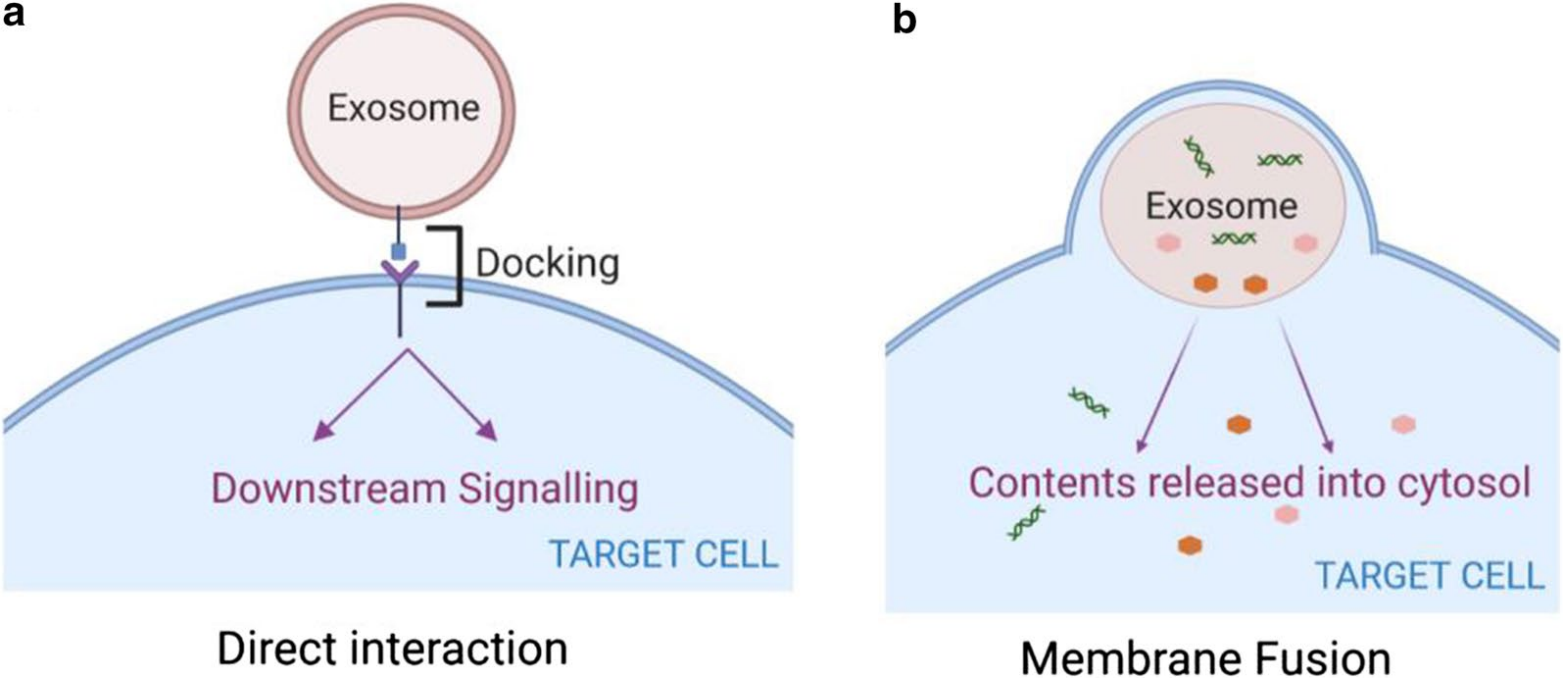
Exosome signalling by direct interaction or membrane fusion. Upon reaching the target cells, **a** membrane receptors within the exosome surface and plasma membrane of target cells can interact inducing downstream signalling cascade in the recipient cell. **b** Exosomes membrane can also fuse with the plasma membrane and release their contents into the cytosol directly

The transmembrane ligands on exosome surface can bind directly with the surface receptors on the recipient cell and generate downstream signalling cascade to activate the target cell (Fig. 4a). This is a common route to mediate immunomodulatory and apoptotic functions. Exosomes released from dendritic cells activate T lymphocytes through MHC-peptide complex [113] and bind Toll-like receptor ligands on bacterial surface to activate bystander dendritic cells and enhance immune responses [114]. Umbilical cord blood-derived exosomes expressing tumor antigens such as MHC-I, MHC-II and tetraspanins (CD34, CD80) also stimultate T cell proliferation to produce antitumor activity [115]. Ligands including tumor necrosis factor (TNF), Fas ligand (FasL) and TNF related apoptosis inducing ligand (TRAIL) expressed on exosome surface released by dendritic cells can bind to TNF receptors on tumor cells and trigger caspase activation for apoptosis [116].

### Fusion with plasma membrane

Exosomes can also fuse with the plasma membrane and release their content directly into the cytosol of target cells (Fig. 4b). This includes hemi-fusion stalk formation between hydrophobic lipid bilayers of the exosome and plasma membrane followed by expansion forming one consistent structure. Families of SNAREs and Rab proteins likely mediate this fusion [117, 118] as shown in studies from cell membrane fusion [119]. Lipid raft like domains, integrins and adhesion molecules present on the exosome surface also mediate interaction, attachment and membrane fusion with the target cell [120–122]. Studies using exosomes incorporating lipophilic dye octadecyl rhodamine B (R18) help distinguishing endocytosis from fusion. R18 is typically introduced into the exosome bilayer at self-quenching concentrations which is diluted upon fusion with unlabelled recipient membranes resulting in concomitant fluorescence, thus allowing to monitor membrane fusion [123]. This process has been observed in dendritic and tumoral cells [124, 125]. Although evidences to support this mechanism remain weak, some authors have speculated that the low pH of tumor microenvironment resulting in higher rigidity and increased sphingomyelin, could facilitate exosome fusion [111], thus making it a likely route to be adopted by tumor cells.

### Internalisation

Exosomes are primarily internalised by the recipient cell followed by cargo release [67, 126, 127]. This uptake process is rapid and temperature-sensitive, decreased by low temperature [105]. The common endocytic pathways are involved in exosome internalisation.

**Fig. 5:**
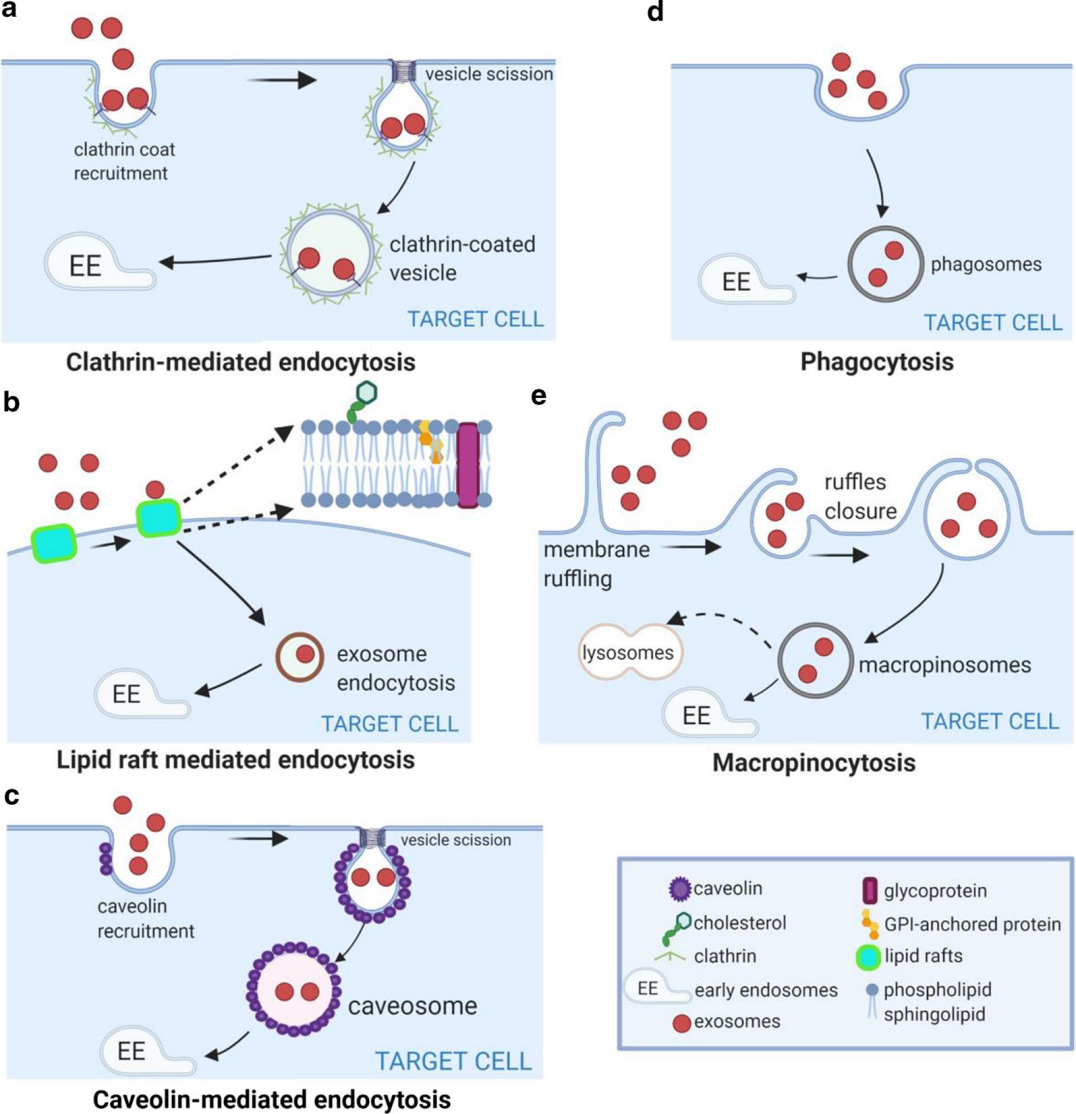
Exosome internalisation. Exosomes are internalised by the recipient cells and fuse with the intracellular compartments/endosomal pathway for cargo release. Exosomes can be internalised by **a** clathrin-mediated endocytosis, **b** lipid-raft mediated, **c** caveolin-mediated endocytosis, **d** phagocytosis or **e** micropinocytosis. These pathways are not always mutually exclusive and can co-exist for the internalisation of a same set of exosomes

Clathrin-mediated endocytosis is a stepwise assembly of various transmembrane receptors and ligands, characterised by the involvement of triskelion scaffold (clathrin), forming clathrin-coated vesicles (Fig. 5a). The internalised vesicles undergo uncoating and fuse with endosomes [128]. This mode of exosome entry occurs in most cell types such as ovarian and colon tumor cells [66, 99, 105], cardiomyocytes [129], macrophages [130, 131], hepatocytes [131] or neural cells [53, 115], epithelial cells [132], illustrated by their dependence on factors essential for clathrin mediated endocytosis. Dynamin-2, an important player in clathrin-mediated endocytosis, forms a collar-like structure in the neck of invaginations required for scission. Dynamin-2 inhibition decreases exosome uptake by macrophages [130, 133] and microglia cells [134]. In cancer cells, the overexpression of transferrin receptor, a major cargo for clathrin-mediated endocytosis, facilitates enhanced exosome uptake [135]. Cumulative evidence suggests clathrin-mediated endocytosis to be one of the canonical exosome uptake pathway. This highly regulated process can also be influenced by the cargo and exosome composition [128].

Lipid raft-associated membrane invagination is a major endocytic mechanism to shift cargo into early endosome (Fig. 5b) and influence exosome uptake [136]. Lipid rafts are detergent-resistant membrane microdomains enriched in cholesterol, sphingolipids and glycosylphosphatidylinositol (GPI)-anchored proteins [121]. Metabolic inhibition of complex lipids alters exosome uptake by lipid rafts. Methyl-β-cyclodextrin, which interferes with intracellular cholesterol transport reduces exosome uptake in breast cancer cells [137]. Exosome uptake by dendritic cells is impaired when the exosome producing cell is pre-treated with a sphingolipid synthesis inhibitor [138]. Pre-treating tumor cells with filipin, which binds to cholesterol and forms ultrastructural aggregates, results in reduced exosome uptake [111, 139]. Annexin AnxA2 promotes lipid raft-mediated endocytosis by immobilising exosomes on the cell surface at specific adherent sites [137]. Flotillin, a component of lipid rafts, also positively regulates lipid raft-mediated endocytosis [140].

Conflicting reports have involved caveolin-dependent endocytosis as another potential exosome uptake route. Caveolin-dependent endocytosis is mediated by integral membrane proteins named caveolins, which create a small flask or omega shaped plasma membrane invaginations called caveolae [141]. Caveolae enable internalisation of caveosomes, large vesicles enriched by highly hydrophobic and detergent-resistant membrane lipids containing cholesterol and sphingolipids (Fig. 5c) [141]. Caveolin-1, 2 and 3 are the main structural proteins of caveolae [141]. Caveolin-1 positively regulates exosome uptake in epithelial cells [142] but negatively regulates exosome uptake in fibroblasts and glioma cells [143]. Both clathrin- and caveolin-mediated endocytosis share molecular players such as dynamin-2, which hinder their differentiation [135] and warrant further studies. This is for instance the case in macrophage activation mediated by exosomal Wnt5a for invasion of breast tumor cells [128, 138]. However, using specific clathrin inhibitors can help differentiate between the two uptake pathways [125].

Phagocytosis typically engulfs large particles like bacteria and dead cells but can also internalise small particles like exosomes. Phagocytosis is a stepwise process where cell membrane deformations encircle the bulk extracellular particles forming phagosomes eventually directing internalised cargo to lysosomes [144] (Fig. 5d). Phosphatidylinositol-3-kinase (PI3K) and phospholipase C (PLC) enzymes are necessary for the phagosome closure. Unsurprisingly, this route of exosome uptake is predominantly used by immune cells such as macrophages and dendritic cells, demonstrated by their dependence on PI3K and actin cytoskeleton activity [124, 130].

Macropinocytosis uses actin-driven lamellopodia to induce inwards plasma membrane invagination that get pinched off to form intracellular compartments called macropinosomes (Fig. 5e). They are growth factors dependent and result in non-specific uptake of extracellular soluble molecules, nutrients and antigens [145]. Cholesterol-mediated Rac1 GTPase recruitment, Na+/H+ exchanger function and in some cases dynamin regulate macropinocytosis [146]. The subsequent macropinosome matures and is then internalised by fusion with the lysosome for degradation or recycling back to the plasma membrane [147, 148]. Exosome uptake can rely on macropinocytosis in HeLa cells [149], subsets of microglial cells [134] highlighted by an Na + /H + exchanger activity dependent uptake [134] and partially in epithelial cells [32]. Anecdotical micropinocytosis, dependent on growth factors, has been reported in Ras-expressing carcinoma cells. The secretion of growth factors could induce micropinocytosis by the use of EGFR stimulation [150]. Uptake of engineered exosomes targeting oncogenic KRAS was also facilitated by RAS-mediated macropinocytosis [151].

These different modes of exosome entry can co-exist. Exosome uptake in ovarian tumor and melanoma cells occurs mainly through-cholesterol associated lipid rafts but clathrin-mediated endocytosis, phagocytosis and micropinocytosis are concomitantly used [105, 152, 153]. Macrophage-derived exosomes use both macropinocytosis and clathrin-mediated endocytosis to penetrate hepatocytes and transfer interferon (IFNƴ) induced resistance to Hepatitis A virus [154]. Clathrin-mediated endocytosis and macropinocytosis are used concomitantly for the uptake of PC12-derived exosomes by bone marrow-derived MSCs [155]. Phagocytosis of exosome by macrophages is also lipid-raft dependent [130]. Similarly caveolin-dependent uptake of exosomes by bone marrow stem cells is also partially mediated by macropinocytosis and membrane fusion [125]. Rarely these routes can play opposite roles as observed in glioblastoma cells, which stimulate and inhibit exosome uptake by lipid rafts and caveolin mediated endocytosis, respectively [143].

Recently a specific filopodial mode of entry has been described in fibroblasts [156]. Filopodia are thin, actin-rich cytoplasmic protrusions that allow cells to probe their environment by increasing cellular surface area and interaction with the extracellular ligands [157]. They can influence various cellular processes including exosome uptake in a manner similar to the uptake of pathogenic bacteria and viruses [158]. Exosomes surf on filopodia at constant speed preceding their internalisation as intact vesicles, while some exosomes encounter laterally moving filopodia with grabbing or pulling motions. This actin-dependent process relies on F-actin dependent retrograde flow [156]. The filopodial motion might happen immediately upstream of the endocytic uptake to facilitate exosome internalisation and adhesion via transmembrane molecules such as integrins possibly acting as coupling receptors [158]. However, whether this filopodial base acting as endocytic hotspot for exosome uptake is specific to fibroblasts, what mediates these filopodial surfing motions and whether they precede or replace other uptake routes are not known.

## Exosomes intracellular signalling

Exosomes which fuse with the plasma membrane release their contents into the cytosol [121] while direct interaction of exosomes with the surface receptors of recipient cells induces downstream signalling cascades [115]. The intracellular fate of exosomes post internalisation follows the typical endosomal pathway, from early endosomes as sorting compartments to acidic vesicles i.e. late endosomes and MVBs, which fuses with lysosomes [111, 159], eventually undergoing degradation. Lysosome targeting requires active transport along the cytoskeleton, a process mediated by the lipid composition [160], SNARE proteins [20] and intracellular pH. Supporting this, motion of exosomes and their cargo along intracellular filamentous structures has been recently confirmed [161]. Exosome membrane lipids are directed to other cellular locations for supposed recycling while transmembrane exosome proteins remain in the perinuclear space suggesting degradation [126, 127].

However, exosome cargoes likely bypasses degradation as various studies demonstrate exosome-mediated functional changes in recipient cells [15, 19]. The gradual acidification through the endosomal compartments can facilitate the exosome cargo function. For instance, exosomes incorporating the pH sensitive latent transforming growth factor (TGF) β-1 are activated in the acidic endosomal environment and induce phenotypic changes in the recipient cell [162]. TGF β-1 cargo is retained in the endosomal compartments during signalling, allowing sustained cellular signalling compared to free TGF β-1 [162] The fusion of endosome and lysosome compartments also allows cytosolic cargo exposure through acidification and in a cholesterol-dependent manner [67]. Some exosome content can also passively diffuse across the cytoplasm, potentially creating an exosome leakage [126, 127].

Endoplasmic reticulum (ER) which is a nucleation site for translation [163], could be a route for lysosomal escape enabling cargo release as ER scanning can occur after exosome sorting into the endosome trafficking circuit [156]. This would be a route of choice for exosomes carrying mRNA and miRNAs to release their cargoes in ER for rapid translation and mediation of altered gene expression. Rab5/Rab7 positive endosomal vesicles interact with ER, highlighting the coupling between endosomal maturation and trafficking [164]. Ultimately exosomes fuse with lysosomes possibly degrading excess cargoes.

Nucleoplasmic reticulum is a sub-nuclear compartment consisting of nuclear associated invaginations penetrating into the nucleoplasm, where the nuclear transfer of exosomes can occur. The nuclear envelope associated invaginations linked with the late endosomes can allow delivery of exosome components into the nucleoplasm and is likely a route for nuclear cargoes [165, 166].

Exosomes are also able to use pathways similar to viruses to avoid lysosomal degradation. In dendritic cells internalised exosomes can bypass lysosomal degradation by being routed to a specialised, surface-accessible CD81 positive LAMP-1 negative intracellular compartment contiguous with the plasma membrane, in a manner similar to HIV-1 particles [138, 167]. However whether this property is specific to a cell type or to the exosomes themselves is not known. Fusion with late endosomes also provide an optimal environment for cargo uncoating and release into cytosol via endosome penetration aided by the high concentrations of anionic lipids in late endosomes. Notably the anionic lipid LBPA, which facilitates the cytosolic entry of viruses and viral vectors [168, 169], also allows exosome fusion with the late endosomes in macrophages, followed by cargo uncoating and potential cytosolic release of contents [154, 169].

Other possible routes that allow exosomal escape from lysosomal degradation include redirection of exosome cargoes from endosomal pathway to trans-Golgi network through retrograde trafficking [170], cargo release into the cytosol through release of partially degraded materials from ruptured endosomal or lysosomal compartments [90] or membrane fusion between exosomes and endosomal membrane [67]. Exosomes can also be redirected back to the plasma membrane from early endosomes via recycling endosomes [171]. This uptake and release cycle possibly allows dissemination to multiple cellular layers and paracrine effect [89].

Hence, exosome cargoes can undertake multiple routes to bypass direct lysosomal degradation to fulfil their signalling functions and the routes can be determined by the cell type, exosome composition and/or the cargoes. It is equally plausible that some exosomes are fated for direct degradation. This seems to be the case in the constitutive macropinocytotic internalisation of oligodendroglial exosomes by subset of microglia lacking antigen-presentation capacity, thereby acting as a mechanism for oligodendroglial membrane clearance in a ‘silent’ manner [134]. Of note, most of the studies determining intracellular exosome fate use labelled exosome membrane lipids and proteins making it challenging to study the cargo fate itself. Despite progress in cargo loading efficacy, direct evidence of cargo release is limited. Labelled membrane bound cargoes combined with high resolution imaging have allowed the detection of cargo exposure in the cytosol of acceptor cells [67]. However, development of better tools to understand the intracellular pathways of exosomes and their cargoes is key to improve our understanding of how exosomes deliver their signalling function.

## Controversies in exosome research

### Exosome biogenesis at plasma membrane

EVs of endosomal origin are identified as exosomes. EVs produced directly from outward plasma membrane budding are classified as ectosomes/MVs and display a size range from 50 nm to 1 µm [2, 37]. Some controversial studies however have suggested that exosome formation can happen directly at the plasma membrane within discrete domains. Plasma membrane of Jurkat T cells have domains enriched in exosome proteins and lipids, referred to as “endosome-like”, potentially to allow rapid and direct exosome biogenesis [172]. Outward vesicle budding from plasma membrane rich in exosomal proteins like CD63 and CD81 can also be observed within these domains [172, 173]. Another study demonstrated exosome markers CD9 and CD81 to bud out fivefold more efficiently from plasma membrane than from endosomal compartments [174]. Still debated, further evidences are necessary to support exosome biogenesis at plasma membrane. Whether this is due to limited characterisation of vesicles studied and/or lack of definite markers to differentiate between different vesicles is also arguable.

### Exosomes heterogeneity and characterisation

Heterogeneity of exosomes due to their varied size, composition, function and cellular origin adds complexity to their characterisation. Distinct exosome subpopulations have been identified, differentiated by their sizes and density [97, 175]. Advanced fractionation separated exosomes by their size, classifying them as large exosomes (90–120 nm) or small exosomes (60–80 nm) [97], while additional density centrifugation separated high and low density exosomes [175]. It is likely that the limiting membrane of MVBs during ILV formation or differences in molecular routes uptaken for exosome biogenesis contribute to these differences [37]. Such heterogeneity can result in differential exosome contents as such the exosome subpopulations are distinct in both their biophysical properties and in their composition [97, 175]. Overall > 4400 proteins, ~ 200 lipids, > 1600 mRNA and > 750 miRNA have been identified from exosomes [176]. Proteomic analysis further reveal that not all exosome proteins are shared among all exosomes regardless of parent cells. Only a small fraction is cell-specific reflecting cell type and physiological condition [177]. Exosome loading varies as reflected by differential qualitative and quantitative content of cargoes [178] influenced by cellular biology and microenvironment [179]. Supporting this, study on cancer cells show differential miRNA packaging by selectively packing tumour inducing miRNAs within exosomes [180]. Cancer cells also secrete higher quantities of exosomes compared to normal cells [181]. Such heterogeneity result in diverse organ biodistribution and distinct biological functions [97, 175, 181].

Recognising exosome heterogeneity is essential to determine their content, functional role and to allow better EV differentiation. Currently isolation methods such as ultracentrifugation, size exclusion, immunoaffinity isolation coupled with analysis methods such as nanoparticle tracking, electron microscopy, flow cytometry and western blots are employed for exosome generation and characterisation [2]. Employment of global and targeted proteomics further aids this process [2]. However, lack of standardisation of these methods has led to substantial overlap in protein profiles of isolated EVs. Lack of specific or universal markers for EVs particularly for MVs and exosomes also complicates their differentiation [2]. Characterisation guidelines placed by the International Society for extracellular vesicles (ISEV) board are being continuously reviewed owing to the evolving nature of EVs and exosome research [182]. Nevertheless to help standardise the field, categories of markers to be analysed in all bulk EV preparations are listed in the Minimal Information for Studies of Extracellular Vesicles (MISEV) guidelines along with recommended changes in reporting of EV terminology [182]. This include classifications based on size (small, medium/large), densities (low, middle, high), biochemical compositions (surface markers) and/or cellular origin [182]. The constant improvement of isolation and purification methods along with continuous research advancements in EV biology is providing increasing support. A recent study highlighted annexin A1, a membrane-associated protein, as exclusive marker for MVs and lack of glycolytic enzymes and cytoskeletal proteins as potential negative markers for exosomes [183]. Having a standard set of markers unique either to the isolation method used or the parental cell is also proposed [2].

### Pitfalls in exosome research

Deciphering exosome biology has been challenged by some pitfalls that the research field aims to address. For instance, molecular players in exosome biogenesis are also involved in other cellular trafficking pathways. Loss and gain of function experiments implemented to study their roles can be exerting direct or indirect effects e.g. altering their function in another cellular vesicular pathways including Golgi, lysosomes and autophagy. This can result in secondary effect on exosome production or secretion [37]. Variation in parent cell types, culture conditions, lack of standardised exosome generation and characterisation methods can all impact experimental reproducibility leading to an overlap in chemical and physical properties between EVs [2, 16]. Implementing multiple, complementary characterisation methods and tracking for any co-isolated non-EV/exosome components is key for better classification [182]. However not all studies implement such rigorous characterisation leading to mixed population of vesicles [177], inadvertently hampering studies on the effect of intended exosomes. Moreover, a survey showed that some researchers have studied the effect of exosomes from culture media rather than intended target cell derived exosomes [184].

Unintended effect of contamination from mycoplasma and other microorganisms, which alters the cellular physiology of donor cells and release their own exosomes, also need to be taken into account [182]. Effect of pre-analytical variables from biofluids and conditioned media need to be explored. In analysing tissues, exosomes either from the extracellular space or artfactual intracellular vesicles released during tissue processing can flaw experimental outcomes [182]. Other factors, such as processing and storage, also alter exosome physiology and affect exosome research [182]. Identifying and overcoming these experimental artefacts are keys for the reliable advancement of exosome research.

### Conclusion

**Fig. 6:**
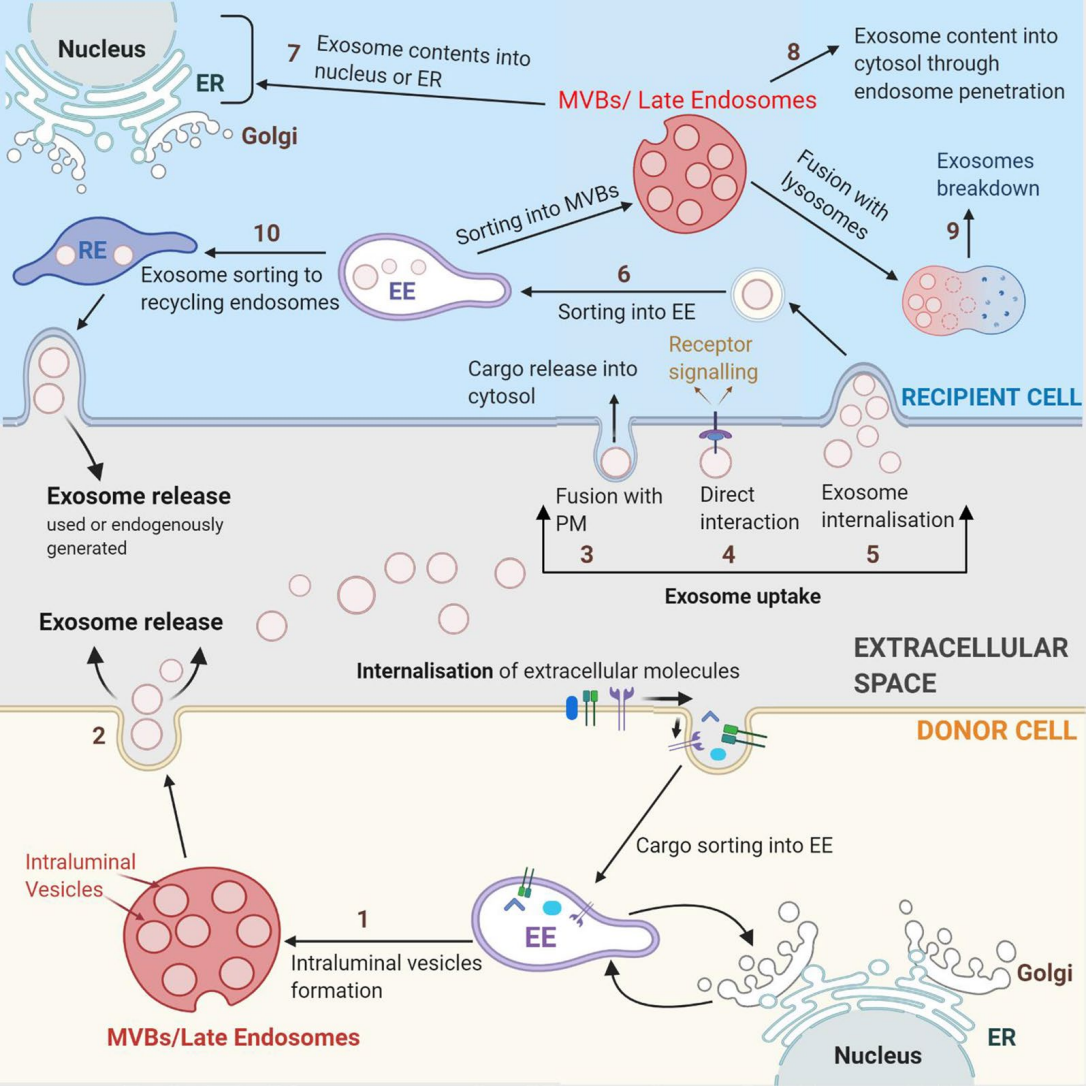
Exosome biology. [1] Exosomes are generated through the formation of ILVs in the late endocytic pathway and [2] gets secreted via exocytosis from the plasma membrane. Upon reaching the target recipient cell, [3] exosomes either interact with the surface molecules of recipient cell to induce downstream signalling or [4] fuse with the plasma membrane to release their contents into cytosol or [5] get internalised via various routes. [6] Upon internalisation, exosomes are addressed in the early endosome, then late endosomes or MVBs and undergo multiple fates. [7] The exosome contents can get released into the nucleus or ER, [8] leak into cytosol or [9] get degraded in the lysosomes. [10] Another possibility include release back to the extracellular space through the recycling endosome. ILV: Intraluminal Vesicles; EE: Early Endosomes, RE: Recycling endosomes, MVB: Multivesicular Bodies, ER: Endoplasmic Reticulum

Studying exosome physiology is a novel and rapidly expanding field of cellular biology. The important role of exosomes in cell–cell communication has been highlighted in multiple studies exploring their physiological and pathophysiological functions. This is essential as these vesicles once secreted can provide key information from the cell of origin similar to a “cell biopsy”. Studies on their clinical application as biomarkers for diagnosis, disease severity and response to therapy along with engineering applications as delivery vectors for therapeutic cargoes are actively being developed and rapidly translated for human applications. These perspectives emphasize the need of a better mechanistic understanding of exosome biology. Various processes and interactions between numerous pathways highlighted in this review provide a framework, which enables delineation of the main steps and routes of interest to enhance cell targeting, exosome uptake or lysosomal escape post internalisation (Fig. 6). If the main mechanisms of exosome biology have been delineated, numerous uncertainties remain about the regulation of these processes. Exosome heterogeneity, their differing content, their properties influenced by donor and recipient cells, lack of standardised exosome characterisation in the literature add to the complexity of unravelling the regulatory processes. Ongoing progress in isolation, characterisation and purification of exosomes in parallel with development of innovative dyes will help in advancing the knowledge of exosome physiology, an essential step for clinical translation of exosome applications.

## Abbreviations

CD: Cluster of differentiation
CHMP: CHarged multivesicular body protein
EBV: Epstein barr virus
EGF: Epidermal growth factor
ESCRT: Endosomal sorting complexes required for transport
EV: Extracellular vesicles
Evi: Evenness interrupted
FasL: Fas ligand
GM: Gangliosides
Hrs: Hepatocyte growth factor-regulated tyrosine kinase substrate
Hsc: Heat shock cognate
Hsp: Heat shock protein
ILV: Intraluminal vesicle
ISEV: International society for extracellular vesicles
LBPA: LysoBisPhosphatidic acid
MHC: Major histocompatibility complex
MISEV: Minimal information for studies of extracellular vesicles
MV: Microvesicle
MVB: Multivesicular bodies
PC: PhosphatidylCholine
PE: PhosphatidylEthanolamine
PI: PhosphatidylInostol
PS: Phosphatidylserine
PI3P: PhosphatidylInositol-3-phosphate
RVG: Rabies viral glycoprotein
STAM: Signal transducing adaptor molecule
SNAP: Soluble NSF attachment proteins
SNARE: SNAP receptor
TfR: Transferrin receptor
TGF: Transforming growth factor
TNF: Tumor necrosis factor
TRAIL: TNF related apoptosis inducing ligand
TSG: Tumor susceptibility gene
